# Supplementary material for: Tissue-dependent mechanosensing by cells derived from human tumors
Source: NPJ Biol Phys Mech. 2025 Aug 6;2(1):19. doi: 10.1038/s44341-025-00023-5 (PMC12328224; doi:10.1038/s44341-025-00023-5)
Supplement: Supplementary file 1 — Supplementary information [file 44341_2025_23_MOESM1_ESM.pdf]

Tissue-dependent mechanosensing by cells derived from human tumors

Kshitiz Parihar[1], Jonathan Nukpezah[2], Daniel V Iwamoto[3], Katrina Cruz[3], Fitzroy J Byfield[3], LiKang Chin[3], Maria E Murray[3], Melissa G Mendez[3], Anne S van Oosten[3], Anne Herrmann[3], Elisabeth E Charrier[3], Peter A Galie[3], Megan Donlick[3], Tongkeun Lee[3], Paul A Janmey[2,3,4], Ravi Radhakrishnan[1,2]

[1]Department of Chemical and Biomolecular Engineering, School of Engineering and Applied Science, University of Pennsylvania, Philadelphia, PA, USA
[2]Department of Bioengineering, School of Engineering and Applied Science, University of Pennsylvania, Philadelphia, PA, USA
[3]Institute for Medicine and Engineering, University of Pennsylvania, Philadelphia, PA, USA
[4]Department of Physiology, Perelman School of Medicine, University of Pennsylvania, Philadelphia, PA, USA

Supplementary Table 1: Number of spread area, aspect ratio, and circularity measurements for each cell line-substrate combination.

	500 Pa Coll	500 Pa FN	30 kPa Coll	30 kPa FN	HA Coll	HA FN	Glass
SK-MEL-2	101	100	100	100	101	100	100
A375	101	100	100	100	100	100	100
WM266-4	100	100	100	100	100	100	100
MeWo	100	96	50	50	100	80	100
RWPE-1(N)	100	103	102	103	101	101	100
22Rv1	99	81	99	100	103	98	103
LnCaP	34	59	41	49	32	46	90
DU145	67	101	77	99	102	103	81
PC-3	100	90	101	100	100	40	100
hTERT-HPNE(N)	106	97	94	100	77	76	100
Panc-1	72	21	100	102	103	105	101
Capan-1	102	100	105	100	101	100	101
SKOV-3	82	104	108	101	100	92	102
Caov-3	37	16	85	34	59	43	81
OVCAR-3	94	89	100	86	100	97	90
NL20(N)	247	284	168	257	126	150	248
NCI-H2126	100	100	100	100	100	100	100
NCI-H2087	100	100	100	100	100	100	100
HCT116	107	101	100	100	100	100	101
HT29	93	40	82	55	95	49	165
SW480	100	100	100	100	100	100	100
SW620	100	100	101	100	100	100	101
hTERT-HME1(N)	101	100	100	100	74	51	100
MCF10A(N)	100	100	100	100	88	62	91
T-47D	100	86	100	100	84	97	100
MCF7	100	82	100	68	45	29	100
MDA-MB-231	145	164	121	218	77	160	193
HCC1937	100	100	100	100	100	100	100
U-87	102	100	101	101	101	100	101
T98G	100	101	100	101	100	101	101

Supplementary Table 2: Number of cell stiffness measurements for each cell line-substrate combination.

	500 Pa Coll	500 Pa FN	30 kPa Coll	30 kPa FN	HA Coll	HA FN	Glass
SK-MEL-2	43	47	45	44	50	43	45
A375	39	44	45	30	42	48	45
WM266-4	42	41	63	44	41	48	44
MeWo	30	33	29	31	33	30	30
RWPE-1(N)	43	48	30	36	45	30	67
22Rv1	45	30	30	30	42	30	45
LnCaP	27	12	29	15	0	38	48
DU145	51	51	47	44	41	50	49
PC-3	35	29	45	39	30	28	45
hTERT-HPNE(N)	50	42	45	49	45	45	45
Panc-1	30	39	36	43	43	44	45
Capan-1	29	27	44	31	32	29	33
SKOV-3	29	27	44	39	30	26	28
Caov-3	44	45	44	45	44	28	45
OVCAR-3	45	44	45	44	47	45	46
NL20(N)	30	33	30	30	30	32	33
NCI-H2126	29	30	30	45	30	30	30
NCI-H2087	32	30	30	29	51	30	30
HCT116	49	42	65	45	66	54	67
HT29	45	32	45	39	45	48	45
SW480	45	59	44	53	45	45	63
SW620	45	32	31	29	30	48	31
hTERT-HME1(N)	30	47	45	45	27	45	47
MCF10A(N)	44	45	45	44	45	45	60
T-47D	45	45	48	44	39	42	48
MCF7	47	42	52	42	45	45	29
MDA-MB-231	30	30	45	45	30	45	32
HCC1937	45	44	42	30	42	42	39
U-87	59	54	51	35	54	45	72
T98G	59	61	63	64	57	56	61

Supplementary Table 3: Number of cell speed measurements for each cell line-substrate combination.

	500 Pa Coll	500 Pa FN	30 kPa Coll	30 kPa FN	HA Coll	HA FN	Glass
SK-MEL-2	61	141	71	72	70	68	129
A375	50	50	50	50	52	51	51
WM266-4	54	51	41	46	51	51	50
MeWo	255	53	176	58	30	72	159
RWPE-1(N)	114	102	119	109	108	105	66
22Rv1	50	45	36	42	50	56	48
LnCaP	29	50	48	50	48	53	50
DU145	27	43	41	11	50	39	32
PC-3	13	99	13	21	24	14	101
hTERT-HPNE(N)	51	51	55	52	47	50	52
Panc-1	33	8	45	44	62	55	57
Capan-1	79	17	52	46	53	30	56
SKOV-3	39	49	33	26	62	44	51
OVCAR-3	49	53	29	52	51	50	58
NL20(N)	54	67	61	70	45	44	26
NCI-H2126	72	75	51	56	56	103	85
NCI-H2087	103	114	87	27	93	69	104
HCT116	44	9	47	36	26	23	70
HT29	57	15	50	36	50	50	50
SW480	55	83	56	50	40	65	0
SW620	51	51	25	25	51	36	25
hTERT-HME1(N)	50	54	51	29	20	32	52
MCF10A(N)	50	60	50	50	50	52	50
T-47D	31	46	30	56	81	27	14
MCF7	37	3	22	15	50	36	50
MDA-MB-231	30	29	27	25	12	26	34
HCC1937	54	50	51	49	53	48	50
U-87	52	102	59	54	58	47	70
T98G	123	94	74	82	75	86	62

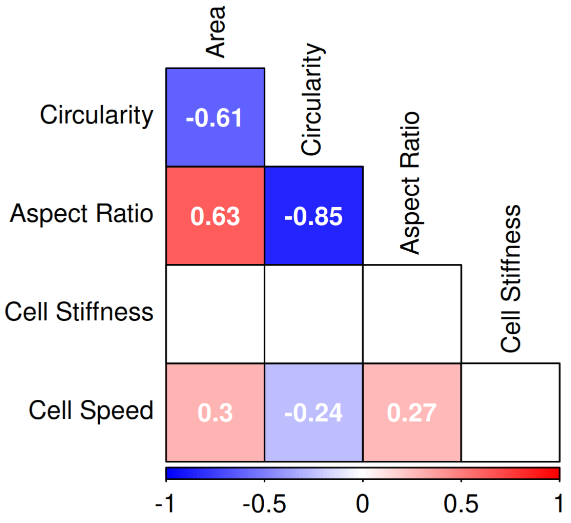

Supplementary Figure 1: Spearman correlation matrix using the median values of the phenotypic features for the cell line-substrate pairs. Only statistically significant correlations are shown. Note that in this analysis only those cell line-substrate pairs are considered which have at least 25 data points for each of the physical feature.

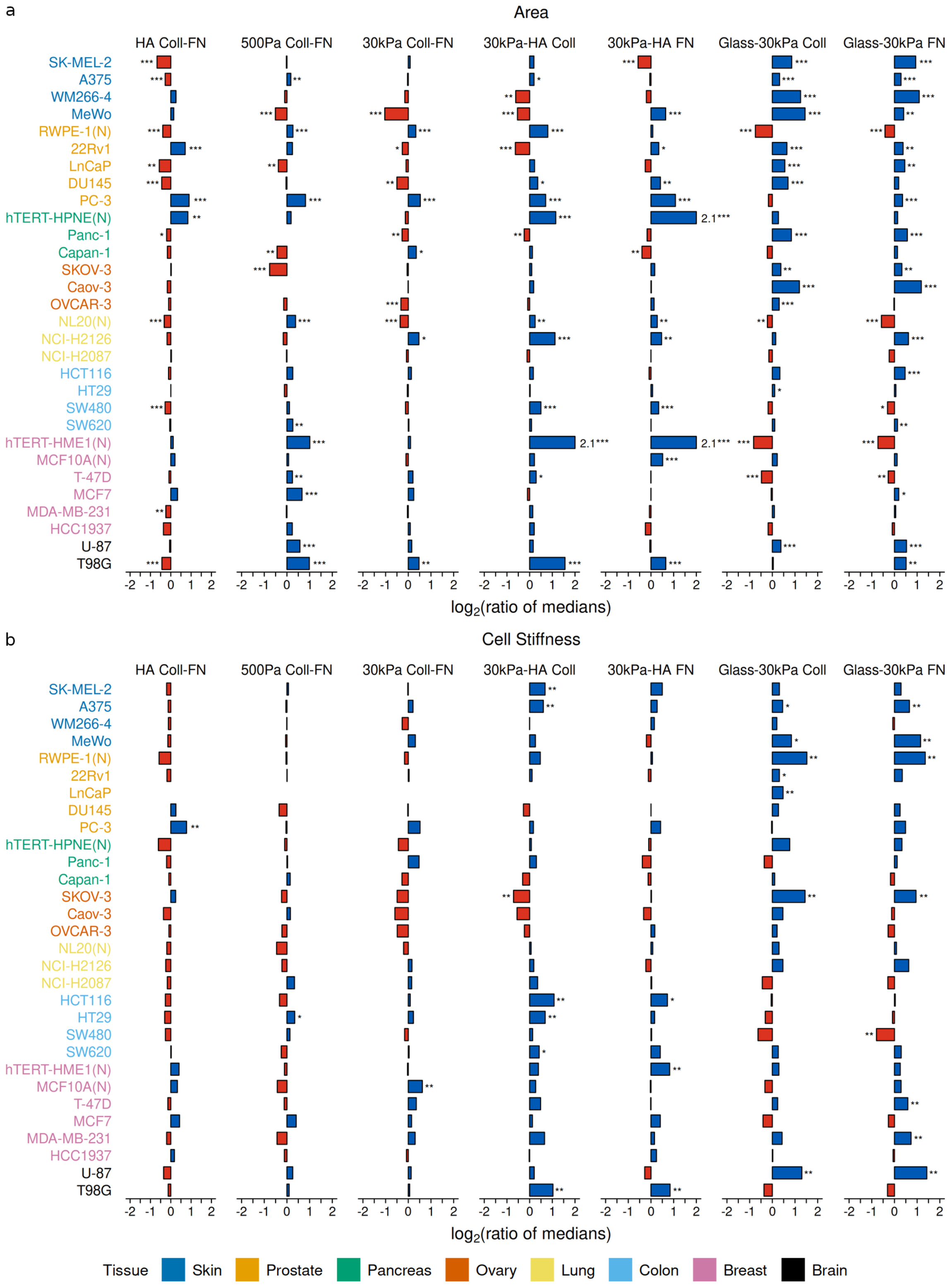

Supplementary Figure 2: For each cell line, the ratio of the median values for (a) area and (b) cell stiffness as a measure of the phenotypic sensitivity to substrate change. (HA Coll-FN: HA Coll/HA FN, 500Pa Coll-FN: 500Pa FN/500Pa Coll, 30kPa Coll-FN: 30kPa Coll/30kPa FN, 30kPa-HA Coll: 30kPa Coll/HA Coll, 30kPa-HA FN: 30kPa FN/HA FN, Glass-30kPa Coll: Glass/30kPa Coll, Glass-30kPa FN: Glass/30kPa FN). (N) refers to non-malignant (normal) cell lines. ***p-value $<$ 0.01; **p-value $<$ 0.05; *p-value $<$ 0.1, adjusted for multiple testing using Benjamini-Hochberg procedure. For each cell line, phenotypic sensitivity to substrate change is calculated only if there are at least 25 data points ($n \geq 25$) for the physical feature of interest on both the substrates. See supplementary tables 1 and 2 for the exact value of n for the cell lines.

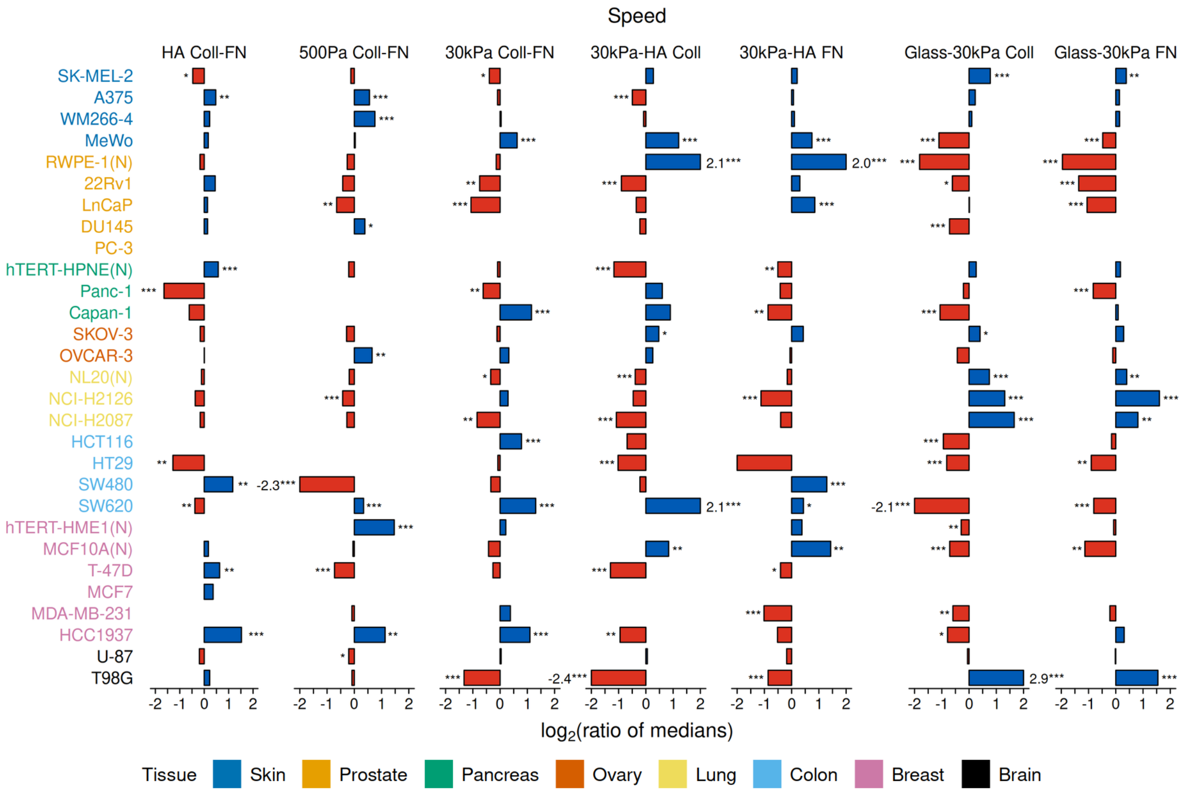

Supplementary Figure 3: For each cell line, the ratio of the median values for cell speed as a measure of the phenotypic sensitivity to substrate change. (HA Coll-FN: HA Coll/HA FN, 500Pa Coll-FN: 500Pa FN/500Pa Coll, 30kPa Coll-FN: 30kPa Coll/30kPa FN, 30kPa-HA Coll: 30kPa Coll/HA Coll, 30kPa-HA FN: 30kPa FN/HA FN, Glass-30kPa Coll: Glass/30kPa Coll, Glass-30kPa FN: Glass/30kPa FN). (N) refers to non-malignant (normal) cell lines. ***p-value < 0.01; **p-value $<$ 0.05; *p-value < 0.1, adjusted for multiple testing using Benjamini-Hochberg procedure. For each cell line on a particular substrate, $n \geq 25$ cells. See supplementary tables 3 for the exact value of n for the cell lines.

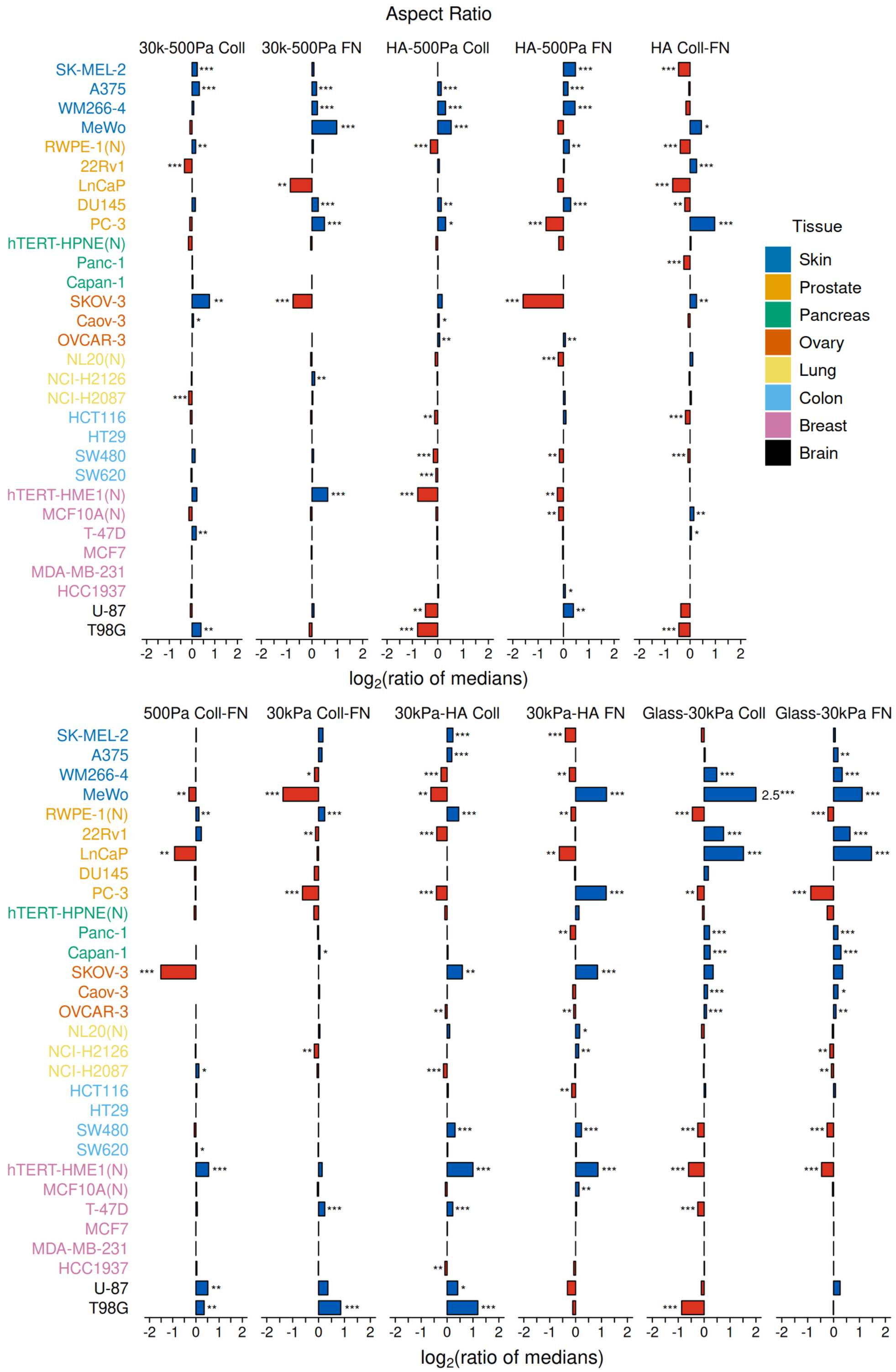

Supplementary Figure 4: For each cell line, the ratio of the median values for aspect ratio as a measure of the phenotypic sensitivity to substrate change. (30k-500Pa Coll: 30kPa Coll/500Pa Coll, 30k-500Pa FN: 30kPa FN/500Pa FN, HA-500Pa Coll: HA Coll/500Pa Coll, HA-500Pa FN: HA FN/500Pa FN, Glass-30kPa Coll: Glass/30kPa Coll, Glass-30kPa FN: Glass/30kPa FN, HA Coll-FN: HA Coll/HA FN, 500Pa Coll-FN: 500Pa FN/500Pa Coll, 30kPa Coll-FN: 30kPa Coll/30kPa FN, 30kPa-HA Coll: 30kPa Coll/HA Coll, 30kPa-HA FN: 30kPa FN/HA FN). (N) refers to non-malignant (normal) cell lines. ***p-value < 0.01; **p-value < 0.05; *p-value < 0.1, adjusted for multiple testing using Benjamini-Hochberg procedure. For each cell line on a particular substrate, $n \geq 25$ cells. See supplementary tables 1 for the exact value of n for the cell lines.

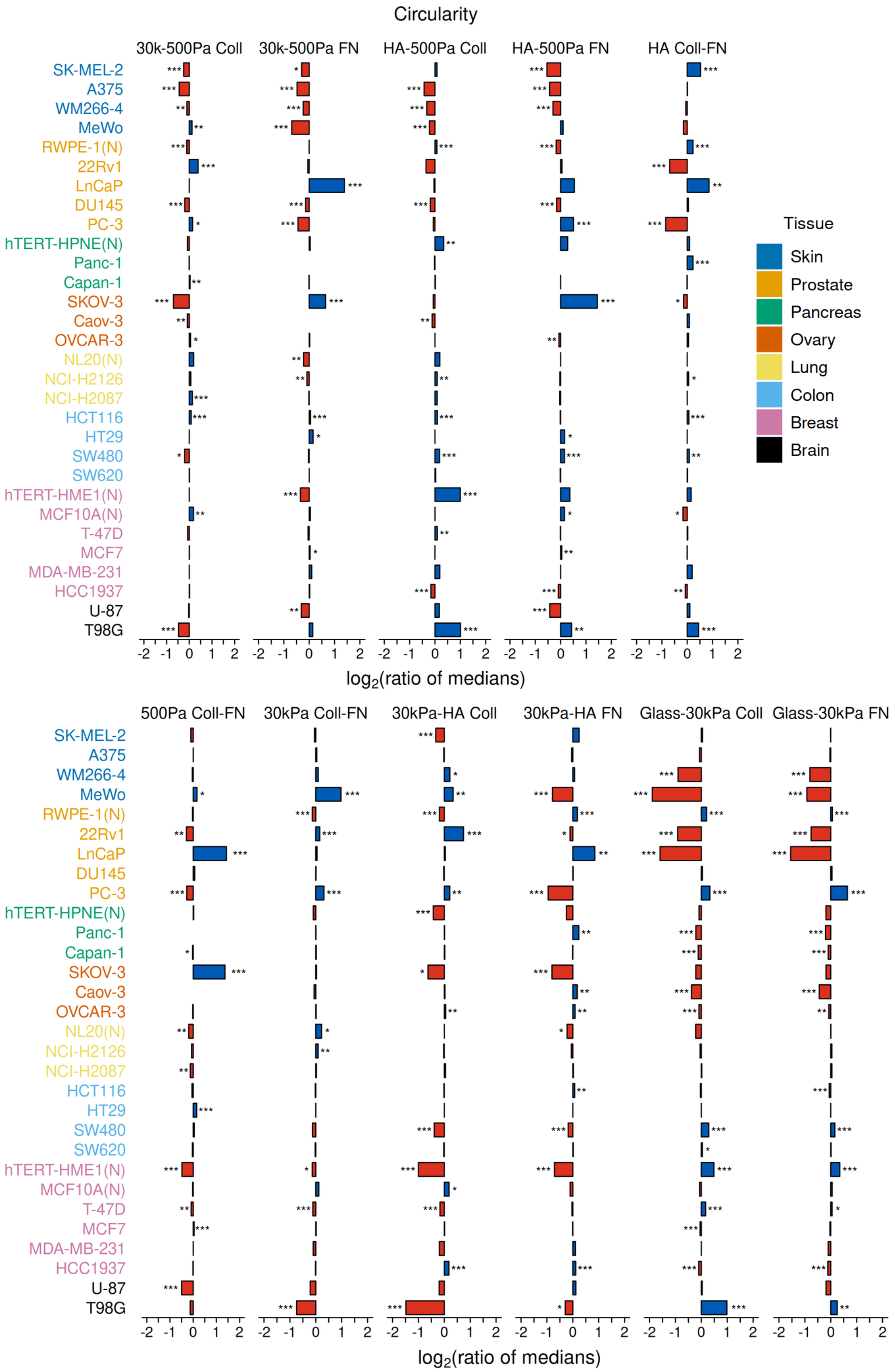

Supplementary Figure 5: For each cell line, the ratio of the median values for circularity as a measure of the phenotypic sensitivity to substrate change. (30k-500Pa Coll: 30kPa Coll/500Pa Coll, 30k-500Pa FN: 30kPa FN/500Pa FN, HA-500Pa Coll: HA Coll/500Pa Coll, HA-500Pa FN: HA FN/500Pa FN, Glass-30kPa Coll: Glass/30kPa Coll, Glass-30kPa FN: Glass/30kPa FN, HA Coll-FN: HA Coll/HA FN, 500Pa Coll-FN: 500Pa FN/500Pa Coll, 30kPa Coll-FN: 30kPa Coll/30kPa FN, 30kPa-HA Coll: 30kPa Coll/HA Coll, 30kPa-HA FN: 30kPa FN/HA FN). (N) refers to non-malignant (normal) cell lines. ***p-value < 0.01; **p-value < 0.05; *p-value < 0.1, adjusted for multiple testing using Benjamini-Hochberg procedure. For each cell line on a particular substrate, $n \geq 25$ cells. See supplementary tables 1 for the exact value of n for the cell lines.

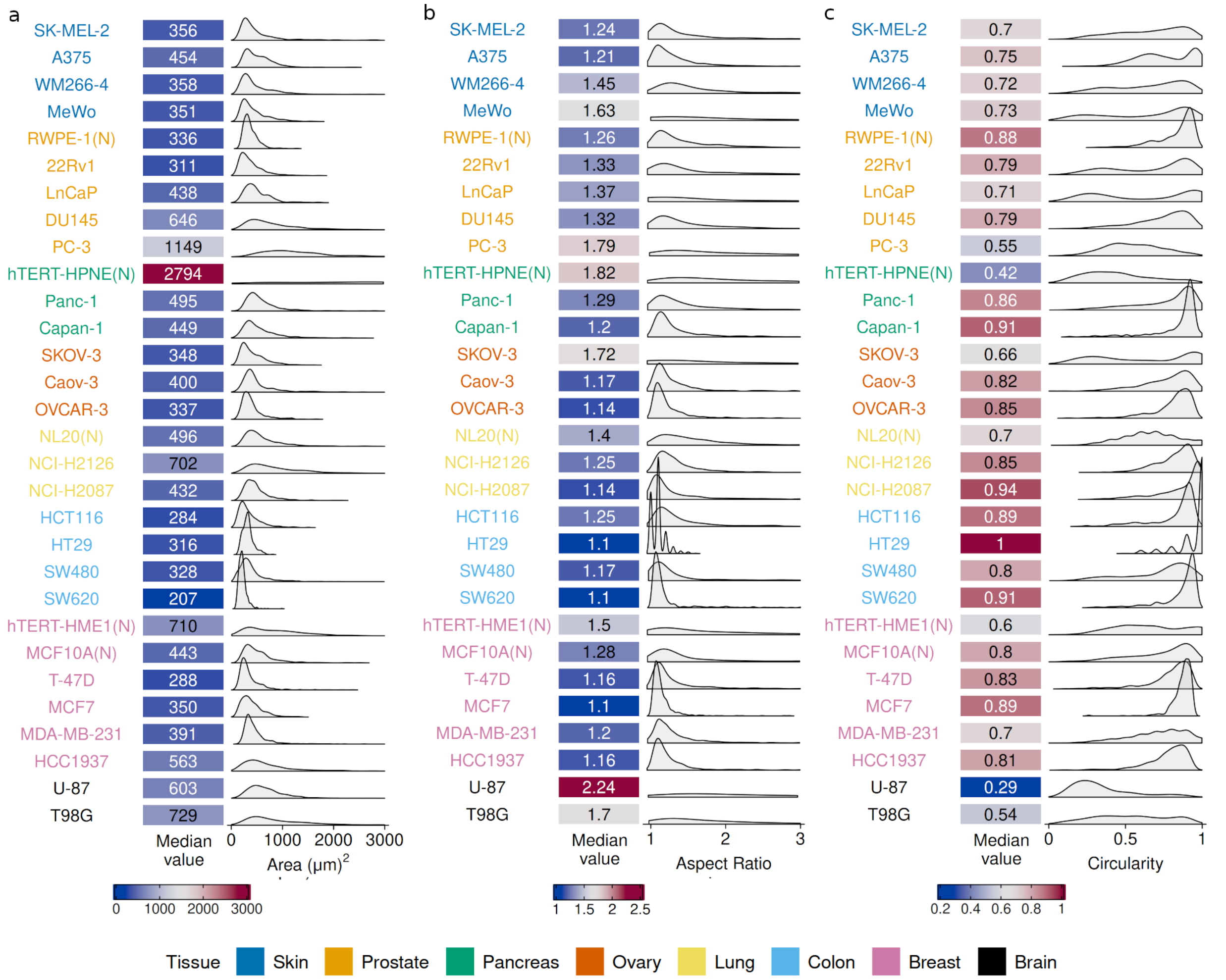

Supplementary Figure 6: Median values of cell (a) area, (b) aspect ratio, and (c) circularity across all the 7 different substrates, along with the kernel density estimates (KDE) for summarizing the distribution of feature values for each cell line. (N) refers to non-malignant (normal) cell lines. See supplementary figure 7c for the KDE of outlier-like distributions of hTERT-HPNE cellular area across all substrates.

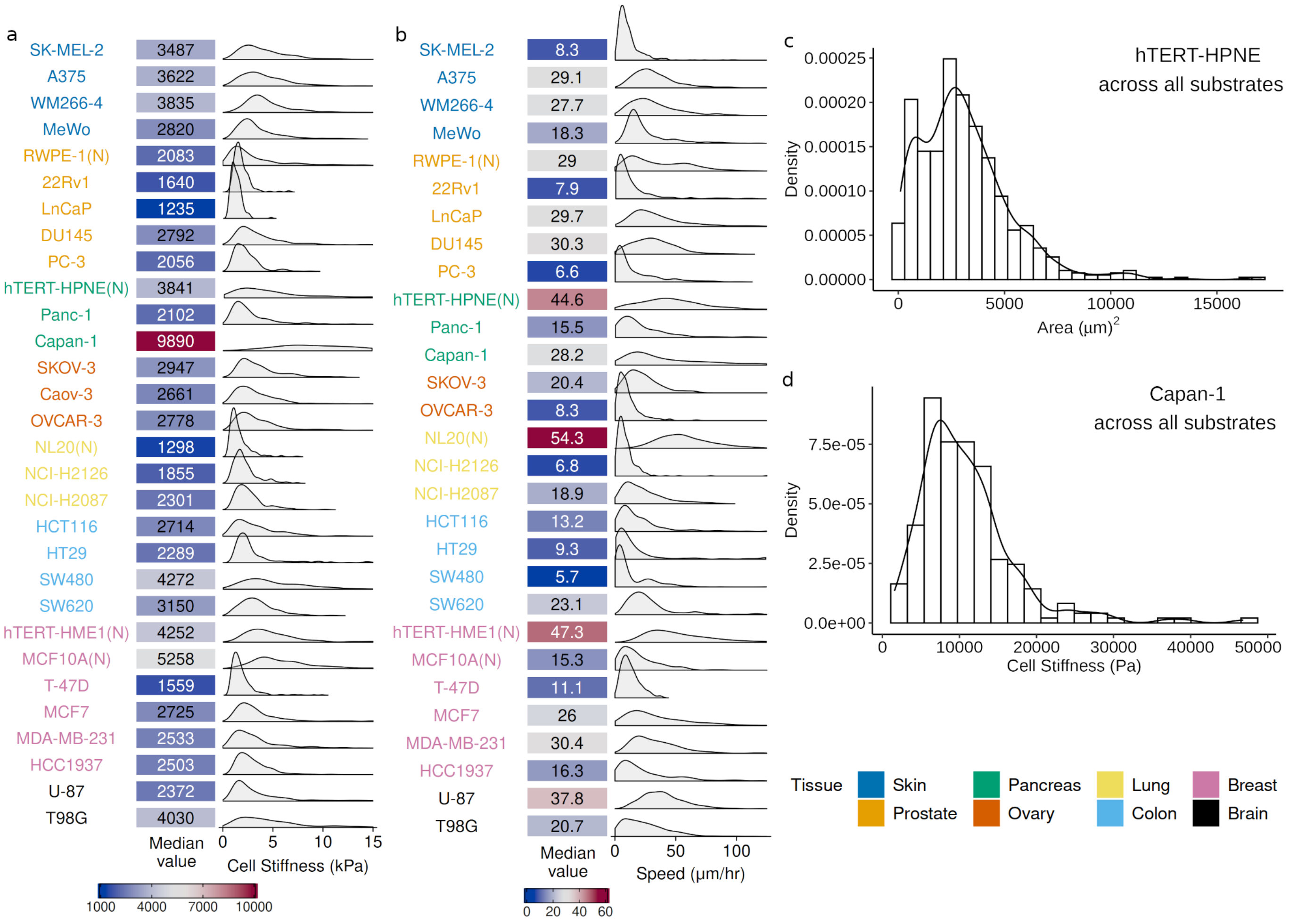

Supplementary Figure 7: Median values of (a) cell stiffness and (b) cell speed across all the 7 different substrates, along with the kernel density estimates (KDE) for summarizing the distribution of feature values for each cell line. (N) refers to non-malignant (normal) cell lines. KDEs for outlier-like distributions of (c) hTERT-HPNE cellular area across all substrates, and (d) Capan-1 cell stiffness across all substrates.

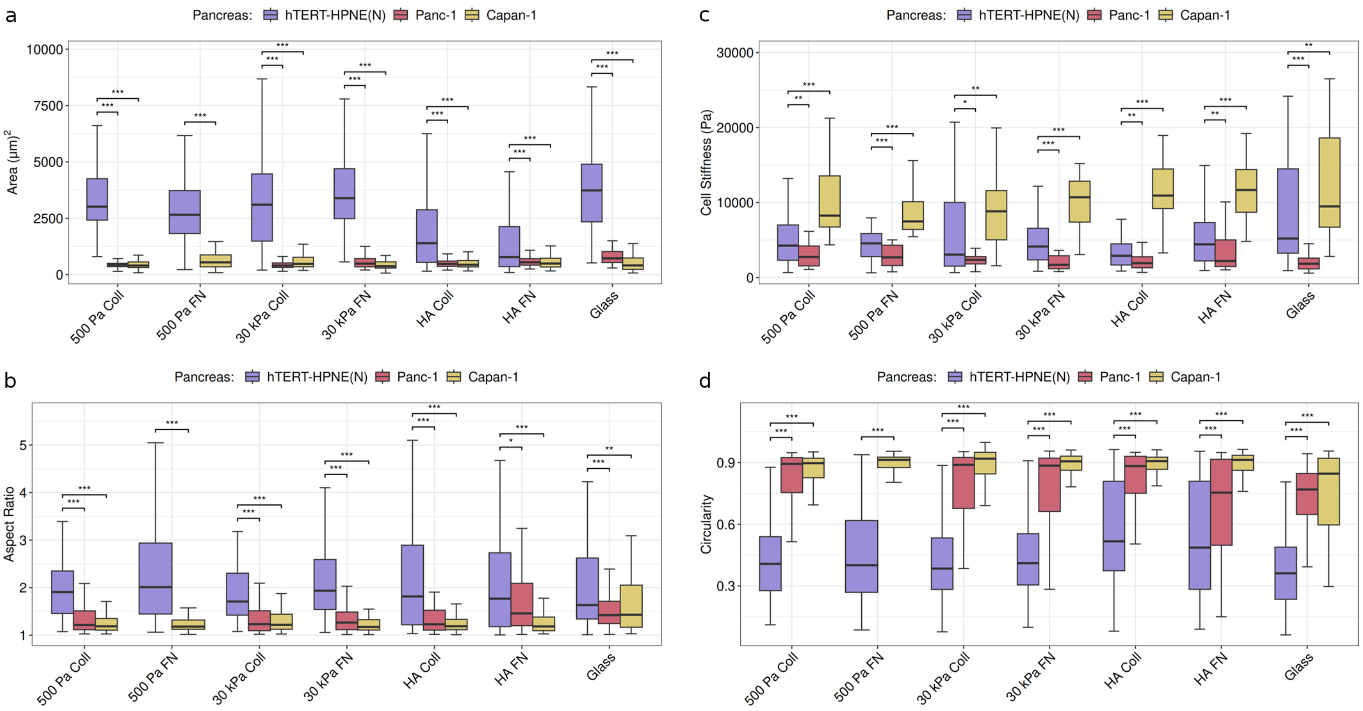

Supplementary Figure 8: Comparing tissue-specific normal and cancer cell behavior in terms of cell (a) area, (b) aspect ratio, (c) stiffness, and (d) speed for pancreatic cell lines soft (500 Pa) and stiff (30 kPa) PAAm Coll and FN substrates, soft (500 Pa) HA substrates coated with Coll and FN, and glass. (N) refers to non-malignant (normal) cell lines. ***p-value < 0.01; **p-value < 0.05; *p-value < 0.1, adjusted for multiple testing using Benjamini-Hochberg procedure. The number of measurements for the physical feature of interest is at least 25 ($n \geq 25$) for each cell line on a particular substrate. See supplementary tables 1-3 for the exact value of n for the cell lines.

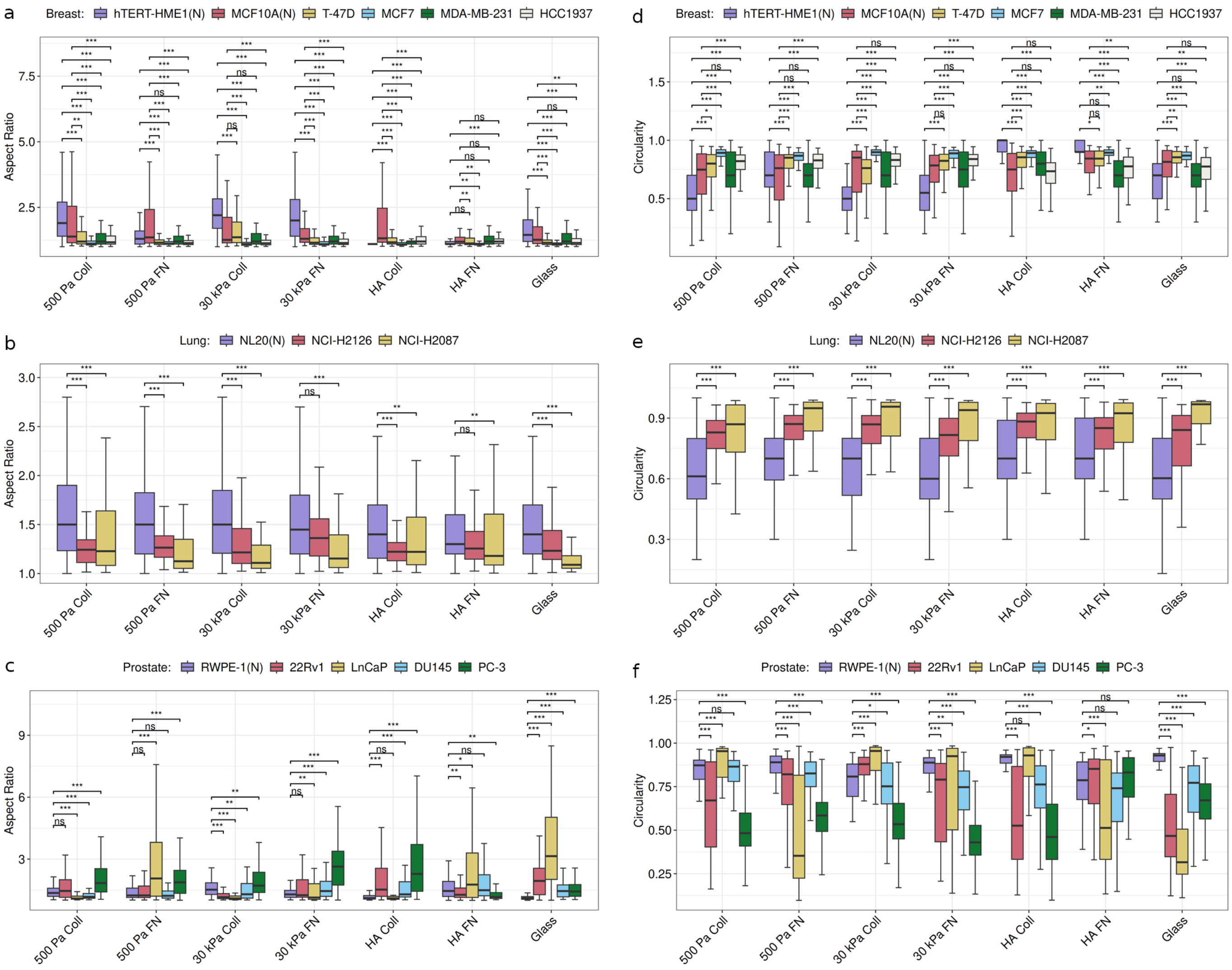

Supplementary Figure 9: Comparing tissue-specific normal and cancer cell behavior in terms of aspect ratio, and cicularity for (a) breast, (b) lung, and (c) prostate cell lines soft (500 Pa) and stiff (30 kPa) PAAm Coll and FN substrates, soft (500 Pa) HA substrates coated with Coll and FN, and glass. (N) refers to non-malignant (normal) cell lines. ***p-value $<$ 0.01; **p-value $<$ 0.05; *p-value $<$ 0.1, adjusted for multiple testing using Benjamini-Hochberg procedure. For each cell line on a particular substrate, $n \geq 25$ cells. See supplementary tables 1 for the exact value of n for the cell lines.

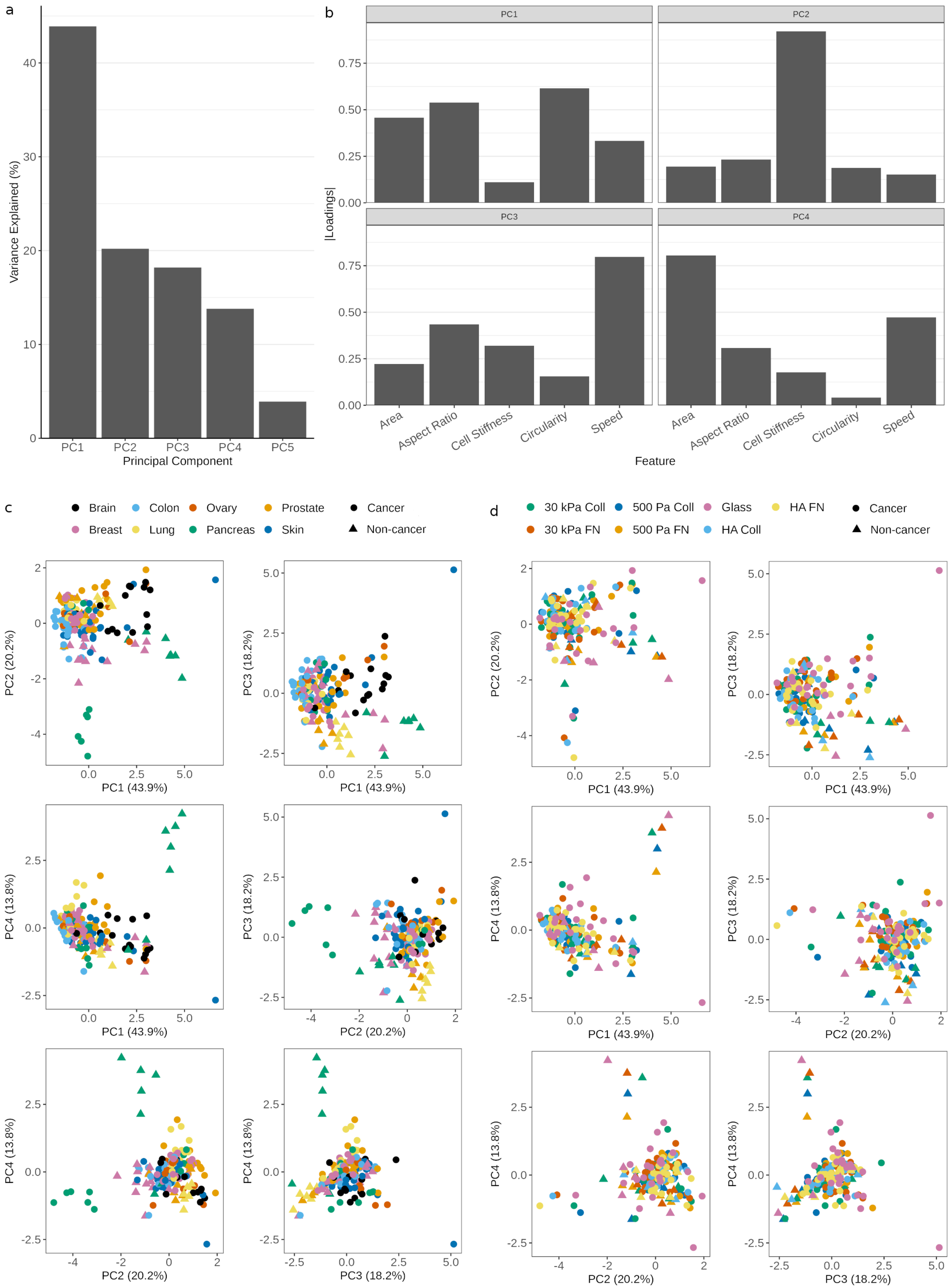
a
Variance Explained (%)
Principal Component
PC1
PC2
PC3
PC4
PC5
b
|Loadings|
Feature
Area
Aspect Ratio
Cell Stiffness
Circularity
Speed
c
Brain
Colon
Ovary
Prostate
Breast
Lung
Pancreas
Skin
Cancer
Non-cancer
PC1 (43.9%)
PC2 (20.2%)
PC3 (18.2%)
PC4 (13.8%)
d
30 kPa Coll
500 Pa Coll
Glass
HA FN
30 kPa FN
500 Pa FN
HA Coll
Cancer
Non-cancer

Supplementary Figure 10: Principal component analysis (PCA) performed using the median values of cell area, aspect ratio, circularity, stiffness, and speedfrom all the cell line-substrate pairs. Note that in this analysis only those cell line-substrate pairs are considered which have at least 25 data points for each of the physical feature. (a) Variance explained by each of the PCs, and (b) the loadings of the physical features on the first four PCs. Pairwise scatter plots for the first four PCs, with the points colored by the (c) tissue type and (d) substrate type.

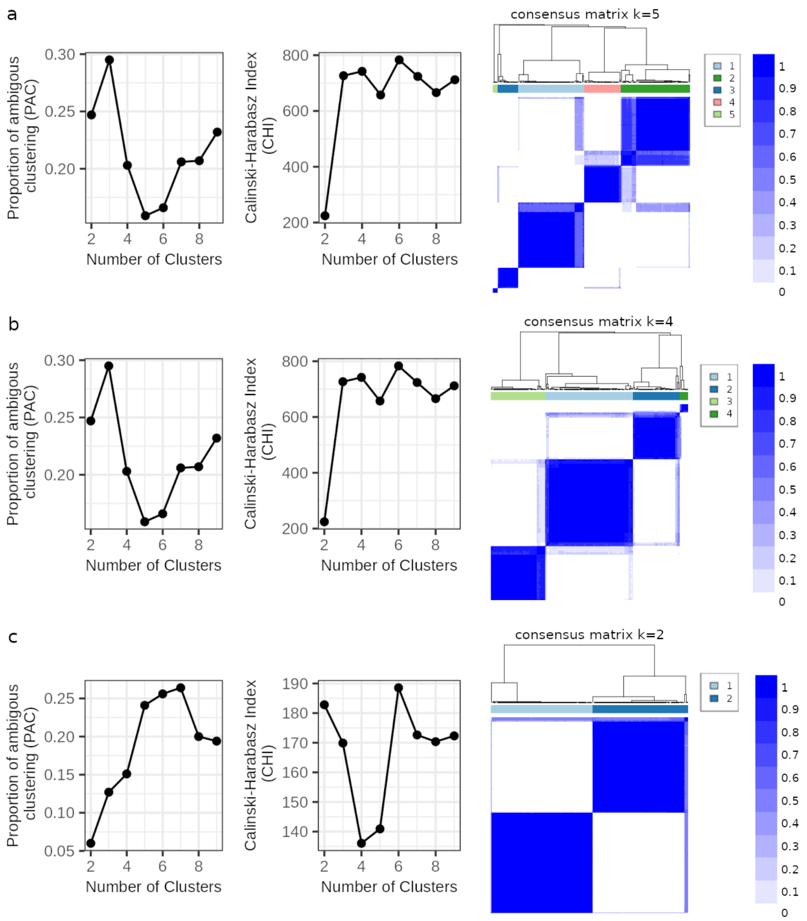

Supplementary Figure 11: Clustering statistics (PAC and CHI) used for identifying the optimal number of clusters (mechanotypes) based on Wasserstein-1 distance and the corresponding consensus matrix for optimal cluster count k of cell (a) area, (b) stiffness, and (c) speed.

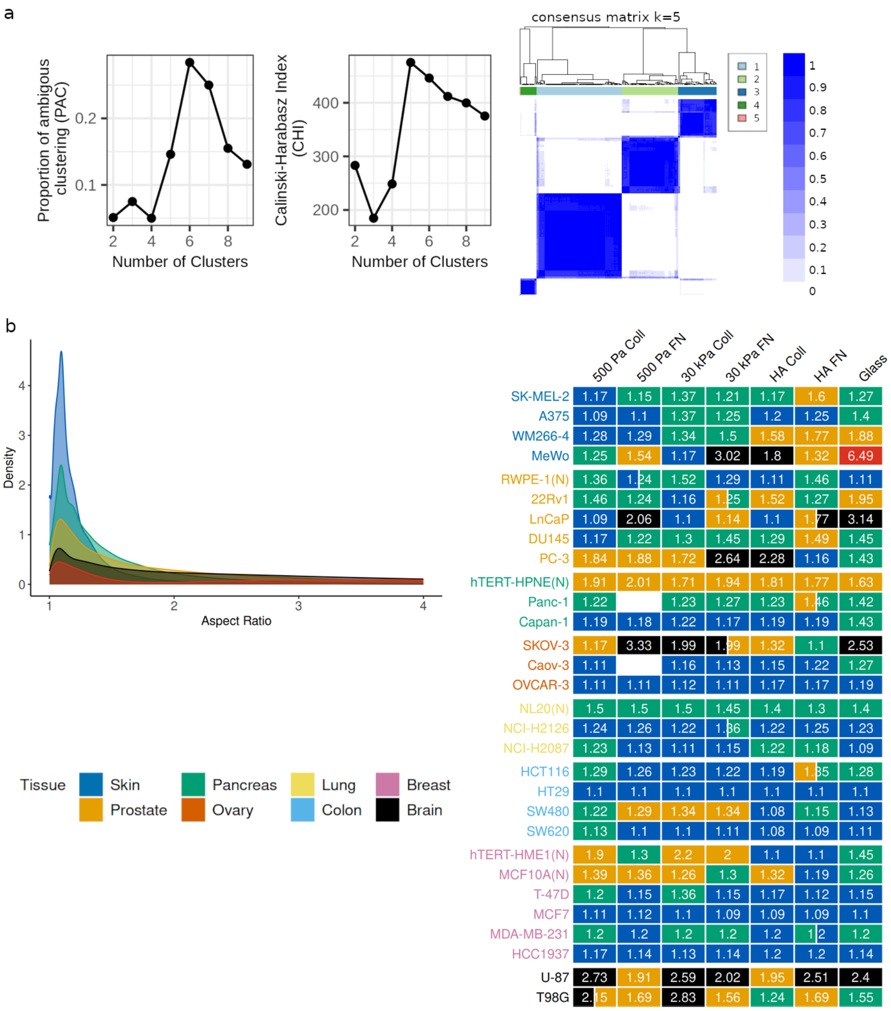

Supplementary Figure 12: (a) Clustering statistics (PAC and CHI) used for identifying the optimal number of clusters (mechanotypes) based on Wasserstein-1 distance and the corresponding consensus matrix for optimal cluster count k of circularity. (b) Mechanotypes for aspect ratio, whereby the heatmap shows the phenotypic class for each cell line-substrate pair and the KDEs correspond to characteristic density function for each class. The numeric values shown in the heatmap correspond to median values of circularity for each cell line-substrate pair. (N) refers to non-malignant (normal) cell lines. Note that in this analysis only those cell line-substrate pairs are considered which have at least 25 data points ($n \geq 25$) for the physical feature of interest. See supplementary tables 1 for the exact value of n for the cell lines.

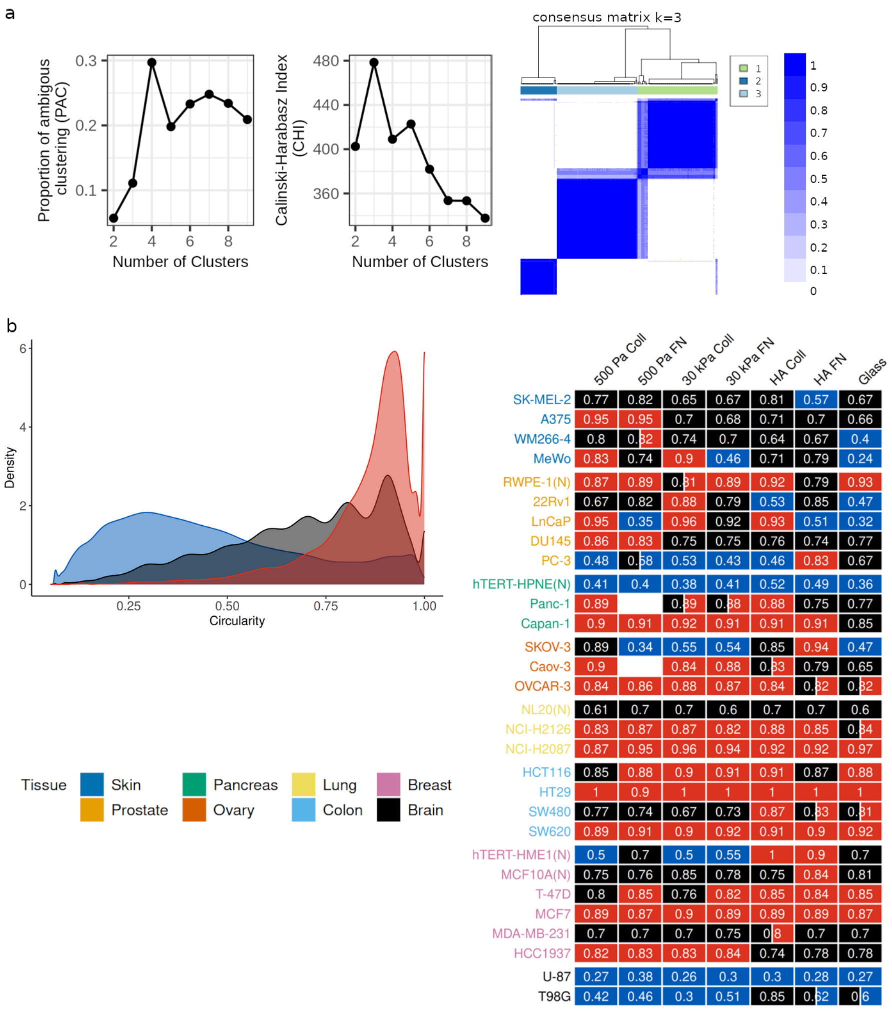

Supplementary Figure 13: (a) Clustering statistics (PAC and CHI) used for identifying the optimal number of clusters (mechanotypes) based on Wasserstein-1 distance and the corresponding consensus matrix for optimal cluster count k of circularity. (b) Mechanotypes for circularity, whereby the heatmap shows the phenotypic class for each cell line-substrate pair and the KDEs correspond to characteristic density function for each class. The numeric values shown in the heatmap correspond to median values of circularity for each cell line-substrate pair. (N) refers to non-malignant (normal) cell lines. Note that in this analysis only those cell line-substrate pairs are considered which have at least 25 data points ($n \geq 25$) for the physical feature of interest. See supplementary tables 1 for the exact value of n for the cell lines.

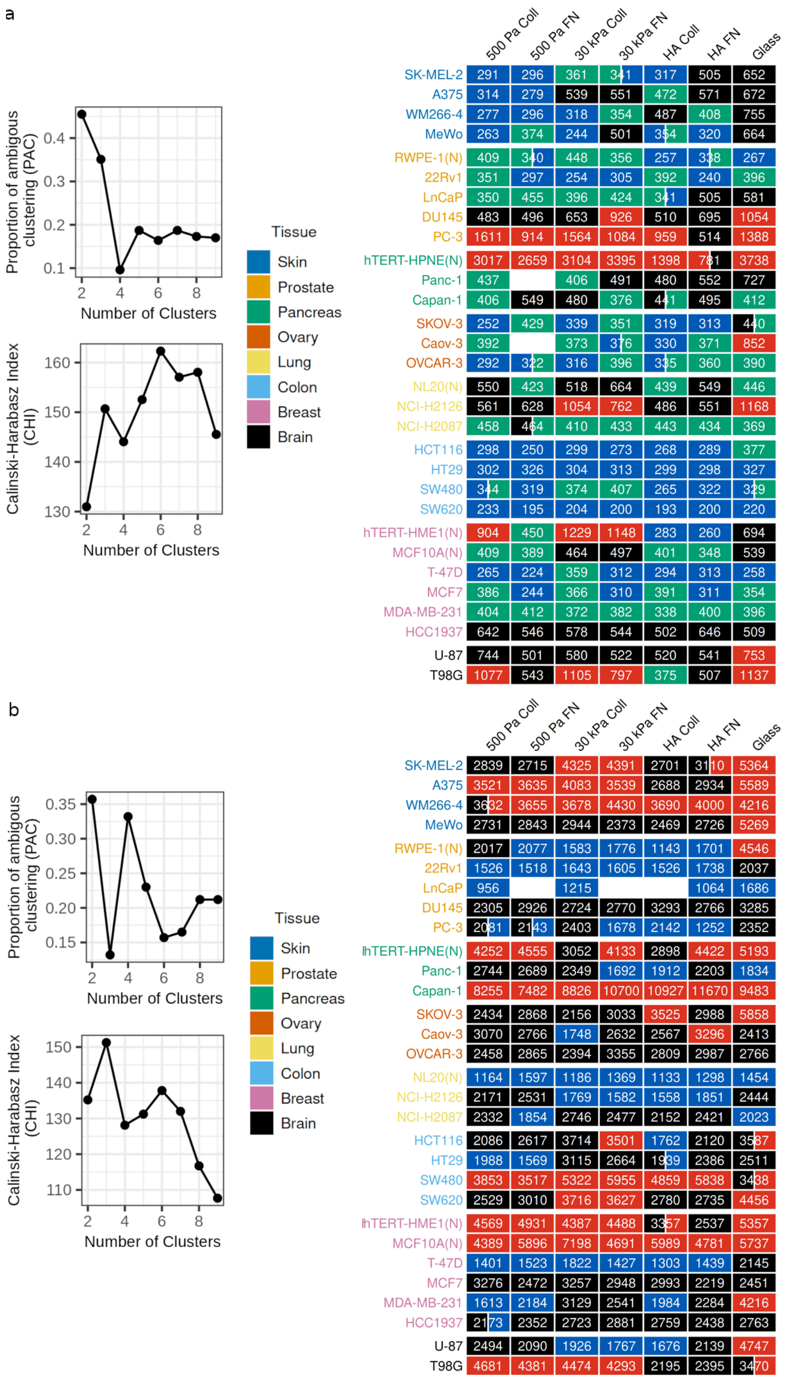

Supplementary Figure 14: Clustering statistics (PAC and CHI) used for identifying the optimal number of clusters based on Kolomogrov-Smirnov distance and the heatmap showing the corresponding classes for each of the cell line-substrate pairs for cell (a) area, and (b) stiffness. The numeric values shown in the heatmap correspond to median values of circularity for each cell line-substrate pair. (N) refers to non-malignant (normal) cell lines. Note that in this analysis only those cell line-substrate pairs are considered which have at least 25 data points ($n \geq 25$) for the physical feature of interest. See supplementary tables 1 and 2 for the exact value of n for the cell lines.

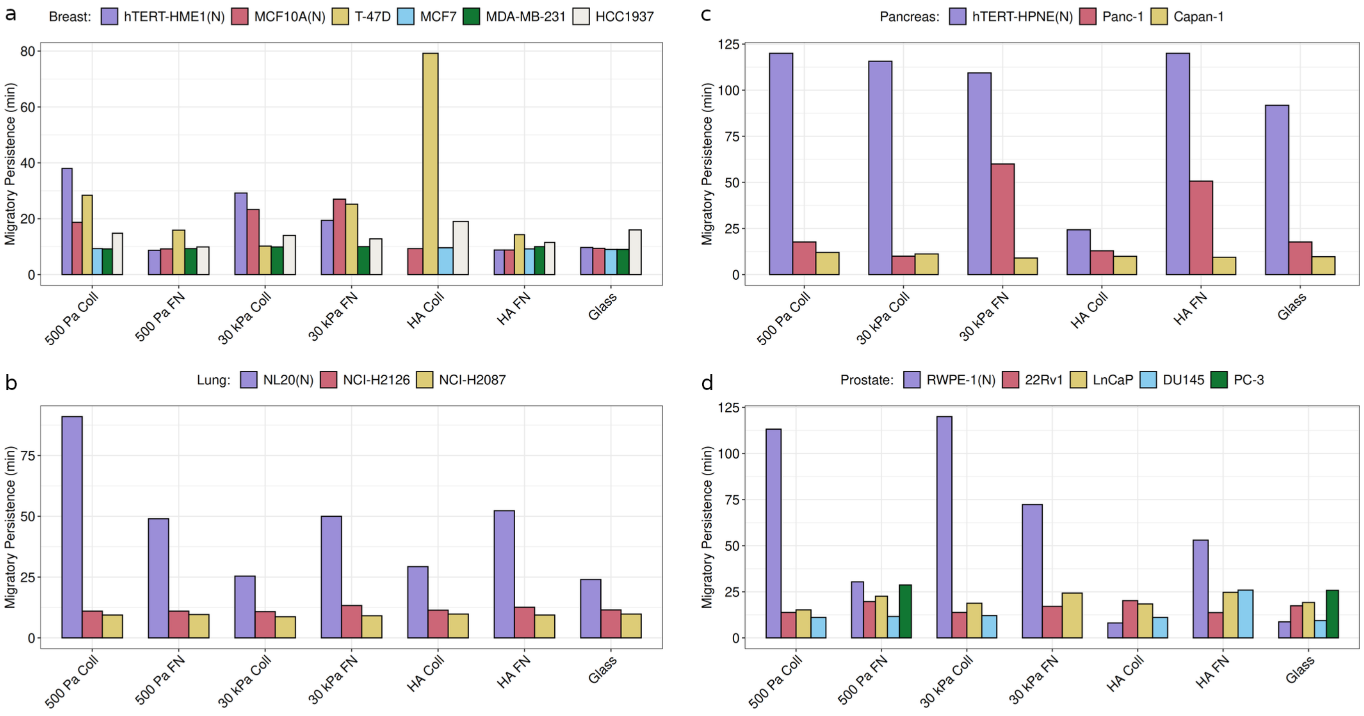

Supplementary Figure 15: Comparison between tissue-specific normal and cancer cell behavior in terms of migratory persistence for (a) breast, (b) lung, (c) pancreas, and (c) prostate cell lines. (N) refers to non-malignant (normal) cell lines. Decorrelation time estimated from directional autocorrelation curves (shown in Supp. Fig. 18-20) is used as a measure of migratory persistence. Note that, for RWPE-1 cell line on 30 kPa Coll, hTERT-HPNE cell line on 500 Pa Coll, 500 Pa FN and HA FN, and Panc-1 cell line on 30 kPa FN, the directional autocorrelation did not decrease below 0.2 cutoff used to determine decorrelation time. For these cases, the total trajectory time is used as a measure of migratory persistence.

	500 Pa Coll	500 Pa FN	30 kPa Coll	30 kPa FN	HA Coll	HA FN	Glass
SK-MEL-2	33.5	38.7	37.7	14.2	23.7	27.4	10.4
A375	21.2	17.8	18.4	20	15.7	18.6	16.8
WM266-4	9.5	9.3	9.6	9.1	12.8	13.2	17.7
MeWo	9.8	8.3	9.7	9.1	9.2	9.2	9.1
RWPE-1(N)	113.2	30.4	120*	72.3	8.1	53	8.7
22Rv1	13.8	19.7	13.8	17.1	20.2	13.7	17.4
LnCaP	15.2	22.6	18.8	24.3	18.4	24.7	19.2
DU145	11.1	11.6	12.1		11.1	25.9	9.4
PC-3		28.7					25.8
hTERT-HPNE(N)	120*	120*	115.7	109.4	24.3	120*	91.8
Panc-1	17.7		10	60*	12.9	50.7	17.7
Capan-1	12		11.2	9	9.9	9.4	9.7
SKOV-3	9.4	44.6	21.9	37.2	11.9	9.6	62.1
OVCAR-3	11	10.9	10.2	19.8	17.6	27.3	14.7
NL20(N)	91	49	25.4	50	29.3	52.3	24
NCI-H2126	11	11	10.8	13.3	11.4	12.6	11.5
NCI-H2087	9.4	9.6	8.7	9.1	9.8	9.4	9.8
HCT116	8.6		9	8.5	8.5		8.4
HT29	62.8		53.6	81.3	9.8	11.2	24.6
SW480	17.8	33.7	8.3	13.5	18.3	10.1	
SW620	9	8.7	9.3	8.9	9.7	9.4	9.4
hTERT-HME1(N)	38	8.7	29.2	19.4		8.8	9.7
MCF10A(N)	18.7	9.2	23.3	27	9.3	8.8	9.4
T-47D	28.4	15.9	10.2	25.2	79.2	14.3	
MCF7	9.3				9.6	9.2	9
MDA-MB-231	9.2	9.3	9.9	10		10	9
HCC1937	14.8	9.9	14	12.8	19	11.5	16
U-87	20.7	24.8	23.6	22.7	40.9	28	29.2
T98G	25.4	25.2	35.9	44.3	28.6	33.3	50.5

Colour scale: 120, 90, 60, 30, 0

Tissue: Skin, Prostate, Pancreas, Ovary, Lung, Colon, Breast, Brain

Supplementary Figure 16: Heatmap showing the migratory persistence for each of the cell line-substrate pairs. (N) refers to non-malignant (normal) cell lines. Decorrelation time estimated from directional autocorrelation curves (shown in Supp. Fig. 17-21) is used as a measure of migratory persistence. Note that (*) represents the cell line-substrate pairs for which the directional autocorrelation did not decrease below 0.2 cutoff used to determine decorrelation time. For these cases, the total trajectory time is used as a measure of migratory persistence.

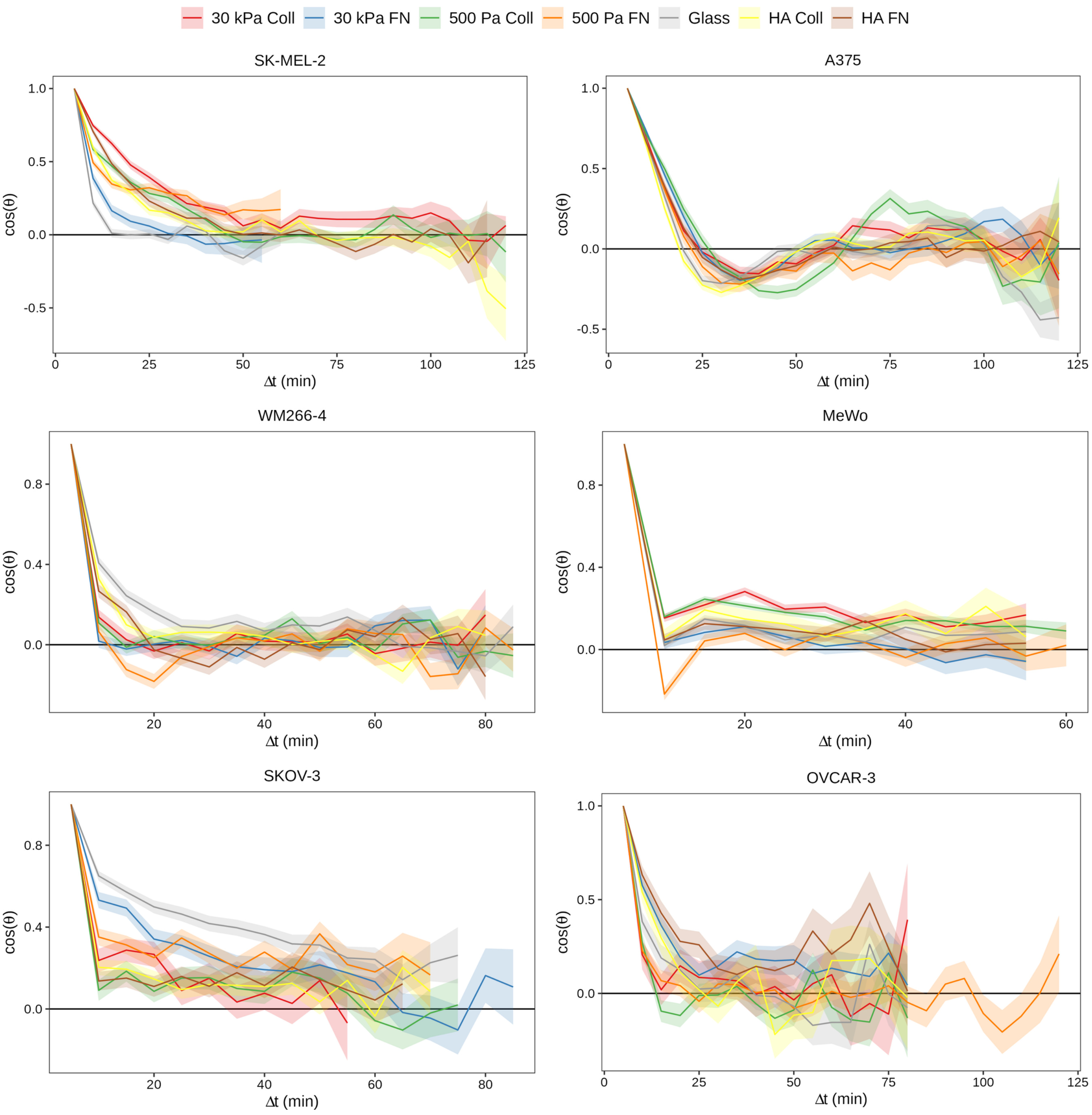

Supplementary Figure 17: Directional autocorrelation comparing the persistence of cell migration for cell lines on different substrates. For each cell line, only those substrates are analysed that have $n \geq 25$ measurements. See supplementary tables 3 for the exact value of n for the cell lines.

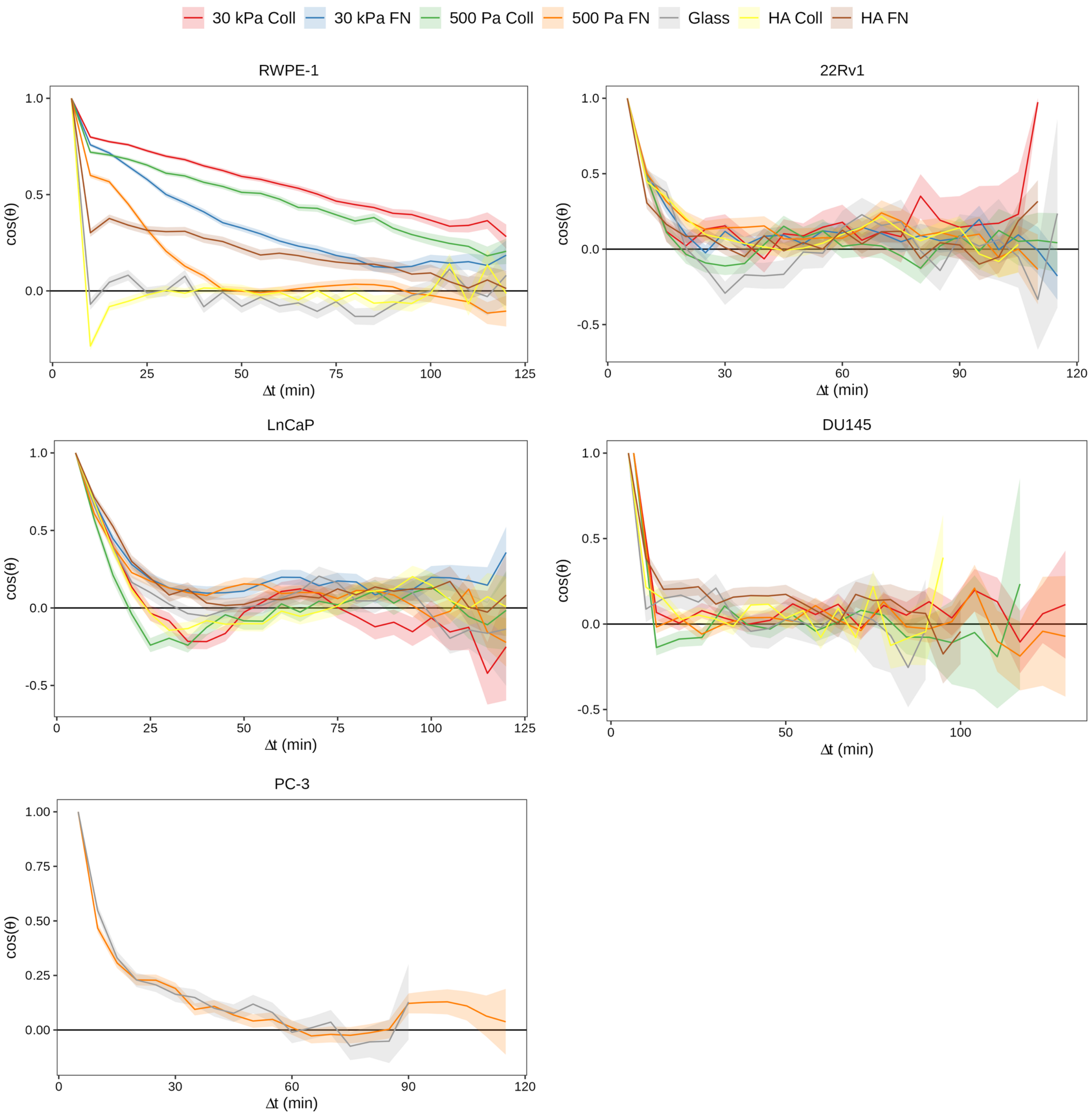

Supplementary Figure 18: Directional autocorrelation comparing the persistence of cell migration for cell lines on different substrates. For each cell line, only those substrates are analysed that have $n \geq 25$ measurements. See supplementary tables 3 for the exact value of n for the cell lines.

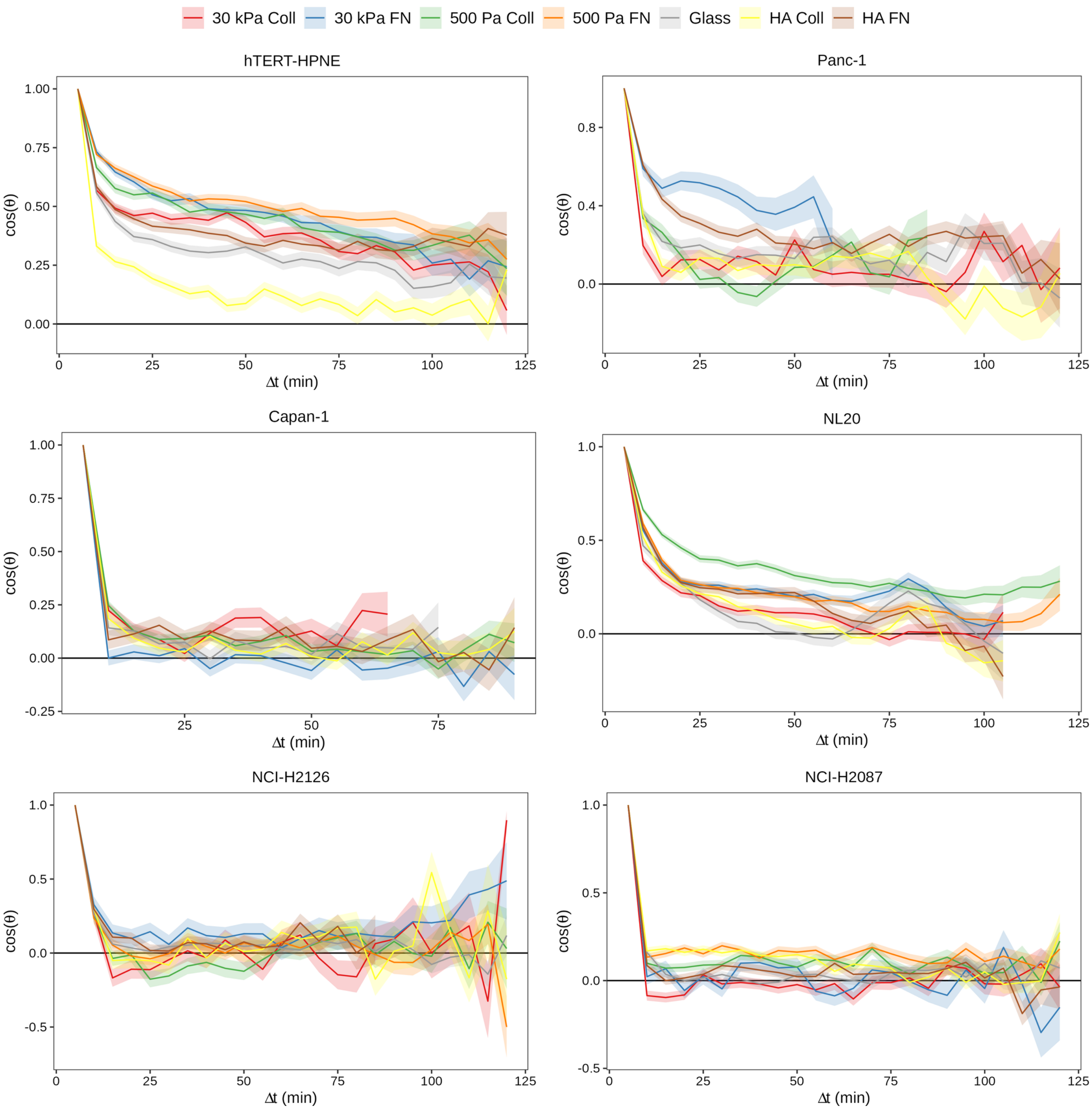

Supplementary Figure 19: Directional autocorrelation comparing the persistence of cell migration for cell lines on different substrates. For each cell line, only those substrates are analysed that have $n \geq 25$ measurements. See supplementary tables 3 for the exact value of n for the cell lines.

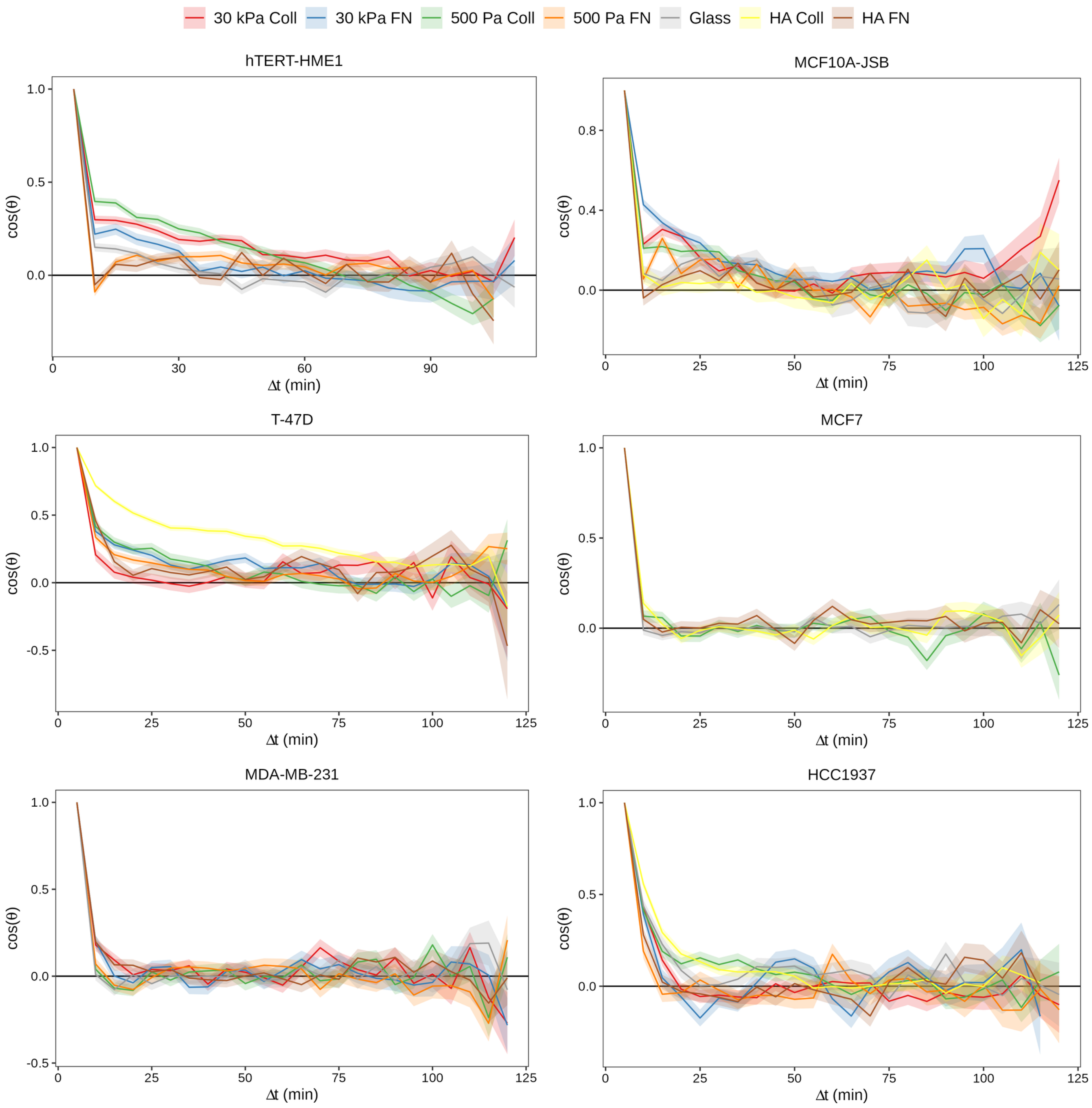

Supplementary Figure 20: Directional autocorrelation comparing the persistence of cell migration for cell lines on different substrates. For each cell line, only those substrates are analysed that have $n \geq 25$ measurements. See supplementary tables 3 for the exact value of n for the cell lines.

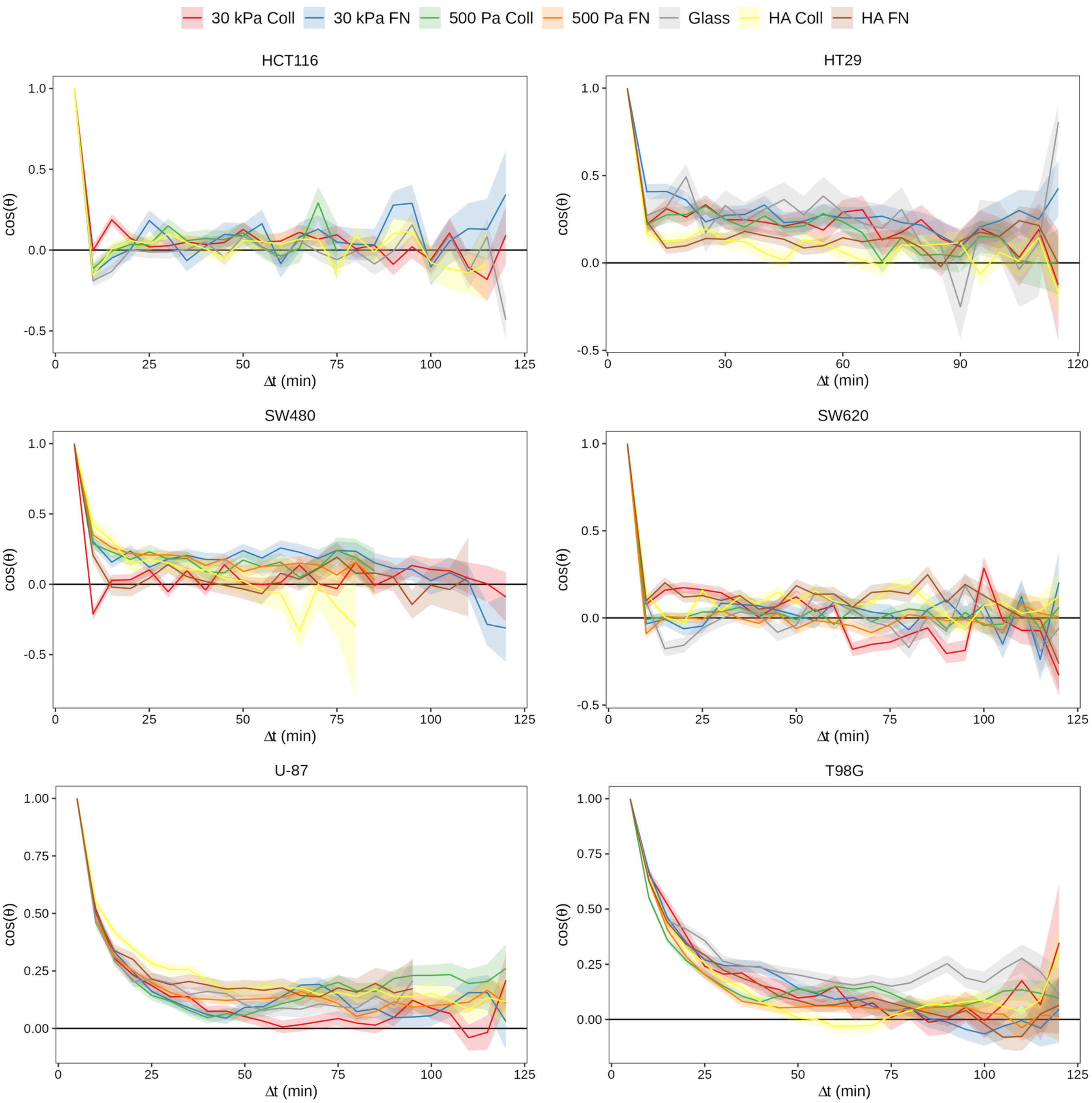

Supplementary Figure 21: Directional autocorrelation comparing the persistence of cell migration for cell lines on different substrates. For each cell line, only those substrates are analysed that have $n \geq 25$ measurements. See supplementary tables 3 for the exact value of n for the cell lines.
